# Bioavailable *Citrus sinensis* Extract: Polyphenolic Composition and Biological Activity

**DOI:** 10.3390/molecules22040623

**Published:** 2017-04-15

**Authors:** Giacomo Pepe, Francesco Pagano, Simona Adesso, Eduardo Sommella, Carmine Ostacolo, Michele Manfra, Marcello Chieppa, Marina Sala, Mariateresa Russo, Stefania Marzocco, Pietro Campiglia

**Affiliations:** 1 Department of Pharmacy, School of Pharmacy, University of Salerno, Via Giovanni Paolo II 132, I-84084 Fisciano, Italy; gipepe@unisa.it (G.P.); fpagano@unisa.it (F.P.); sadesso@unisa.it (S.A.); esommella@unisa.it (E.S.); mchieppa@unisa.it (M.C.); msala@unisa.it (M.S.); smarzocco@unisa.it (S.M.); 2 Department of Agriculture, Laboratory of Food Chemistry, University of Reggio Calabria, Via Melissari stecca n°4, I-89122 Reggio Calabria, Italy; mariateresa.russo@unirc.it; 3 Department of Pharmacy, University of Naples Federico II, Via D. Montesano 49, I-80131 Napoli, Italy; ostacolo@unina.it; 4 Department of Science, University of Basilicata, Viale dell’Ateneo Lucano 10, I-85100 Potenza, Italy; michele.manfra@unibas.it; 5 National Institute of Gastroenterology “S. de Bellis”, Institute of Research, I-70013 Castellana Grotte, Italy; 6 European Biomedical Research Institute of Salerno, Via De Renzi 3, I-84125 Salerno, Italy

**Keywords:** *Citrus sinensis* polyphenols, antioxidant, anti-inflammatory, gastro-intestinal digestion, bioavailability

## Abstract

*Citrus* plants contain large amounts of flavonoids with beneficial effects on human health. In the present study, the antioxidant and anti-inflammatory potential of bioavailable polyphenols from *Citrus sinensis* was evaluated in vitro and ex vivo, using the murine macrophages cell line J774A.1 and primary peritoneal macrophages. Following simulated gastro-intestinal digestion, the in vitro bioavailability of *Citrus sinensis* polyphenolic extract was assessed using the human cell line Caco-2 grown as monolayers on a transwell membrane. Data demonstrated a relative permeation of its compounds (8.3%). Thus, the antioxidant and anti-inflammatory effect of polyphenolic *Citrus sinensis* fraction (Cs) was compared to the bioavailable one (CsB). Results revealed that *Citrus* extract were able to reduce macrophages pro-inflammatory mediators, including nitric oxide, iNOS, COX-2 and different cytokines. Moreover, the effect of *Citrus sinensis* polyphenols was associated with antioxidant effects, such as a reduction of reactive oxygen species (ROS) and heme-oxygenase-1 (HO-1) increased expression. Our results provide evidence that the bioavailable polyphenolic constituents of the *Citrus* *sinensis* extract accumulate prevalently at intestinal level and could reach systemic circulation exerting their effect. The bioavailable fraction showed a higher anti-inflammatory and antioxidant potential compared to the initial extract, thus highlighting its potential nutraceutical value.

## 1. Introduction

Inflammation is a biological process mainly supported by the immune cells to protect the host from microbial infection or tissue injury. Although inflammation is an essential response to eliminate aggressors, its development has to be controlled over time to monitor its intensity and avoid deleterious chronic inflammatory response [1].

The oxidative response is deeply involved in the regulation of the inflammatory processes, mediating many aspects of inflammatory-induced tissue damage and dysfunctions [2,3]. Nowadays, the study of oxygen-containing free radicals in humans and their role has been a growing interest among scientists. Synthetic antioxidants are known to have free radical inhibition properties in the human body, but these compounds could also prove toxic and hazardous to the human body [4]. In this regard, the area of natural products is of great interest. Many studies have recently focused their attention on the potential health effects of polyphenols from different natural sources, such as anticancer and anti-inflammatory properties as well as the ability to reduce oxidative stress [5,6,7]. Plants rich in certain polyphenols have been traditionally used for their anti-inflammatory properties and attention has been given to isolated compounds as potential anti-inflammatory and as natural antioxidant agents. Moreover, different studies have observed the inverse correlation existing between the risk of cardiovascular diseases and polyphenols intake [8]. Most of these effects have been highlighted by the use of in vitro screening methods. Nevertheless, to achieve any effect, polyphenols should reach a specific tissue or organ, a complex process involving extraction from the food matrix and absorption from the gut via the intestinal cells. In addition, gastrointestinal (GI) digestion conditions may result in drastic structural changes that could affect the stability and bioactivity of food constituents [9]. It has been demonstrated, for instance, that the digestion process decreased the phenolic content by at least 47% in digested fruit beverages compared to undigested ones [9]. Tagliazucchi et al. [10], found that only 62% of originally present polyphenols in grapes were bioaccessible after GI digestion, and that radical-scavenging activities of polyphenols was strongly reduced. *Citrus* is one of the largest species among plants; it consists of 40 species which are distributed in all continents and its fruits, which are consumed mostly fresh, have been used as a herbal medicine or additive or food supplement [11]. A wide range of potential health benefits has been ascribed to *Citrus* such as anticancer, antimicrobial, antioxidant and anti-inflammatory [12], deeply associated with its flavonoids content [8]. Since sufficient intestinal absorption is a prerequisite for polyphenols bioactivity, recent studies focused the attention on the bioacessibility of different citrus food matrices, also looking for a correspondence with the biological activities [13,14]. Similar efforts were dedicated to the assessment of *Citrus* flavonoids bioavailability [15,16,17]. On the contrary, the effect of intestinal absorption on the total *Citrus* flavonoidic profile and on their antioxidant and anti-inflammatory effect after GI digestion has not been investigated so far.

For these reasons, in the present study we focused on the antioxidant and anti-inflammatory activity of the bioavailable fraction from *Citrus sinensis* polyphenols using an extended in vitro model, including Caco-2 cell permeation of the intestinal digesta. The polyphenolic profile of *Citrus* at the different stages of simulated digestion was compared to those natively present, while the impact of GI digestion on the antioxidant and anti-inflammatory properties of the *Citrus* extract was determined using an *in-cell* inflammation model.

## 2. Results

### 2.1. Qualitative and Quantitative Polyphenolic Profile of Citrus sinensis

Identification was carried out on the basis of standard retention time, UV spectra, and by comparing molecular formulas generated by the analysis of accurate MS and MS/MS spectra with those present in literature [18,19]. Nineteen compounds were identified (see Supplementary Information, Table S1), belonging to different subclasses such as C and O glucoside flavones, flavanones, flavonols and polymethoxyflavones, which are characteristic of sweet orange and detected in other *Citrus* species [20]. As it can be appreciated from Table S1, the most abundant compounds were the flavanones narirutin and hesperidin in accordance with previous observations reported in the literature, followed by the C-glucosidic compounds vicenin-2 and lucenin-2 4′-methyl ether. Among polymetoxyflavones, nobiletin was the most abundant compound [18,19].

### 2.2. Gastro-Intestinal Digestion and Bioavailability Assessment of Citrus sinensis Juice Flavonoids

On average, flavonoids of *Citrus sinensis* juice extract were detected in the salivary phase at levels ranging from 75.1% to 99.2% of their native patterns, thus evidencing a good stability to the biochemical conditions in the mouth (Table S1). It is well known that the oral mucosa can promote the bioavailability of a wide range of both polar and hydrophobic compounds that rapidly reach the blood circulation bypassing the GI system [21]. Moreover, since a large part of nutrients and non-nutrients are gastro-sensitive and/or poorly absorbed in the intestinal tract, the salivary extraction and the absorption through the oral mucosal epithelium would allow bioactive compounds to target specific tissues and organs without undergoing the potentially degrading effect of the GI digestion and/or being excreted in the feces [21].

**Figure 1:**
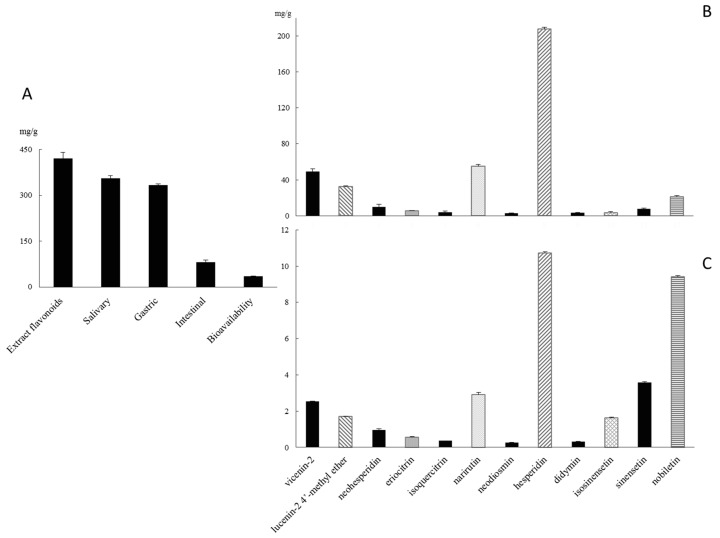
Total polyphenols content in *Citrus sinensis* extract after the in vitro gastro-intestinal digestion process (Panel **A**); Comparison of the polyphenol profile of Cs (*Citrus sinensis*, Panel **B**) and CsB (bioavailable *Citrus sinensis*, Panel **C**).

The gastric phase (treatment with pepsin and the acidification to pH 2, Figure 1A) revealed a good stability of flavonoids to the gastric acidic medium and this step did not increase flavanone precipitation (Table S1). In fact, flavonoid glycosides appear quite resistant to acid hydrolysis in the stomach and consequently reach the intestine intact without forming corresponding aglycones [22]. This was confirmed by Gil-Izquierdo et al. [23] who studied the effect of gastric digestion on the flavonoid glycoside content of orange juices produced by different processing techniques.

Our experimental results confirmed that flavonoid degradation might occur primarily in the intestine (Figure 1A) [24]. On average, a loss of 48.2% and 85.8% of the native flavonoid pattern after intestinal digestion was obtained (Table S1). It has been previously demonstrated that the alkaline conditions of the small intestine, when combined with pancreatin and bile digestion, transform some of the flavanones (hesperidin and narirutin) into insoluble chalcones [23].

The bioavailability of *Citrus sinensis* juice flavonoids was evaluated by using confluent monolayer of Caco-2 cells as a model of absorption in the small intestine. Data reported in Figure 1C, show the aliquots of permeated flavonoids. Generally, most of the glycosides can be absorbed in the small intestine after being hydrolysed by specific enterocyte enzymes [22]. Particularly, polyphenols linked to a rhamnose moiety must reach the colon and be hydrolysed by rhamnosidases before absorption. This is the case of neohesperidin, eriocitrin, narirutin, neodiosmin, hesperidin and didymin, for which an availability range of 5.1–9.7% of their native patterns has been revealed (Figure 1B,C). Only specific glucosides would be absorbed in the small intestine, transported into enterocytes by the sodium-dependent glucose transporter SGLT1 [22]. Extracellular hydrolysis of glucosides by brush-border-membrane hydrolases has been proposed, which is followed by diffusion of the aglycones across the brush border [25]. *Citrus sinensis* juice flavonoid glucosides, such as vicenin-2, lucenin-2,4′-methyl ether and isoquercetrin, revealed an availability of 5.15%, 5.22% and 8.87%, respectively, of their native pattern (Figure 1B,C).

Finally, our data confirmed the good availability of polymethoxyflavones owing to the lipophilic nature of their multiple methoxy groups that contribute to their high permeation across the membranes [26]. These data underline that experiments conducted by using semipermeable cellulose membranes to assess the bioavailability of these types of polyphenols may overestimate their degree of intestinal absorption [27]. Specifically, dialysis bags simulate the intestinal passive diffusion which, albeit a fundamental way of transport through the enterocyte bilayer, is one of the main mechanisms for the cellular polyphenol uptake, together with facilitated diffusion and active transport [22].

### 2.3. Citrus sinensis and Bioavailable Fractions Did Not Affect Cell Viability

To evaluate if Cs or CsB administration could affect J774A.1 or mouse peritoneal macrophages viability, cells were exposed to *Citrus sinensis* extract (Cs) or with the bioavailable extract (CsB) in the culture media. Our data indicated that cellular viability was not significantly affected either by Cs or CsB (data not shown).

### 2.4. Effect of Citrus Extracts on LPS-Induced NO, iNOS and COX-2

**Figure 2:**
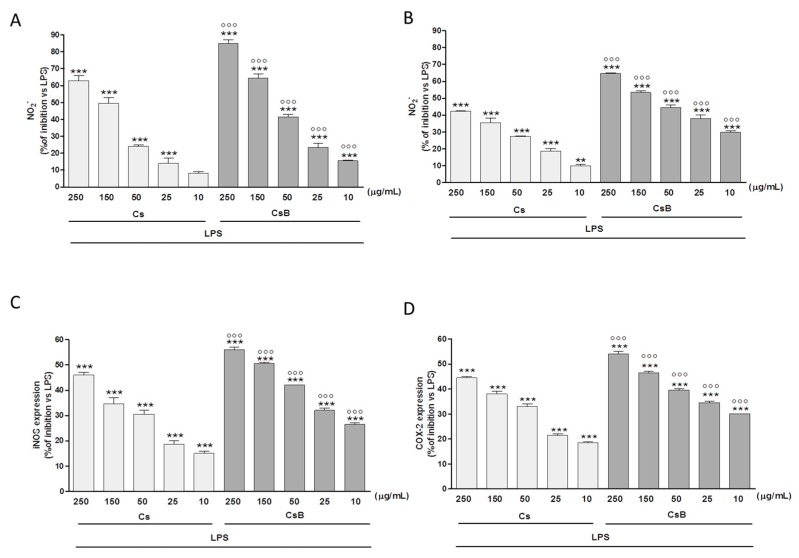
Effect of Cs and CsB (250–10 μg/mL) on NO release, evaluated as NO_2_^−^ (µM), by macrophages J774A.1 stimulated with LPS (Panel **A** and Panel **B**); Effect of Cs and CsB (250–10 μg/mL) on LPS-induced iNOS (Panel **C**) and COX-2 (Panel **D**) in macrophages J774A.1. Values, mean ± s.e.m., are expressed as % of inhibition vs. J774A.1 treated with LPS alone. *** and ** denote *p* < 0.001 and *p* < 0.01 vs. LPS alone. °°° denotes *p* < 0.001 vs. Cs.

In our experimental model, macrophages exposed to Cs significantly reduced NO secretion in a dose dependent manner (250–25 µg/mL, *p* < 0.001 vs. LPS). At the same concentrations CsB proved to be significantly more effective than Cs (*p* < 0.001; Figure 2A). In particular, when added 24 h after LPS treatment, both Cs and CsB inhibited NO release by J774. A1 macrophages at all tested concentrations (*p* < 0.001 vs. LPS), indicating their inhibitory effect both on the iNOS enzyme expression and on its activity. CsB, also in these experimental conditions, was more effective in comparison with Cs (*p* < 0.001; Figure 2B). Similarly, Cs and CsB (250–10 µg/mL) inhibited iNOS expression at all tested concentrations (*p* < 0.05 vs. LPS; Figure 2C). In particular, CsB significantly inhibited iNOS expression at all tested concentrations both versus LPS and versus Cs (*p* < 0.001; Figure 2C). An interaction between iNOS and COX pathway represents an important mechanism for the modulation of the inflammatory response. Our data show that also COX-2 protein expression was strongly inhibited at all tested concentrations by Cs and CsB (*p* < 0.001 vs. LPS). Moreover, COX-2 appeared to be more strongly inhibited by CsB (*p* < 0.001 vs. Cs; Figure 2D).

### 2.5. Effect of Citrus Extracts on LPS-Induced TNF-α and IL-6 Production

**Figure 3:**
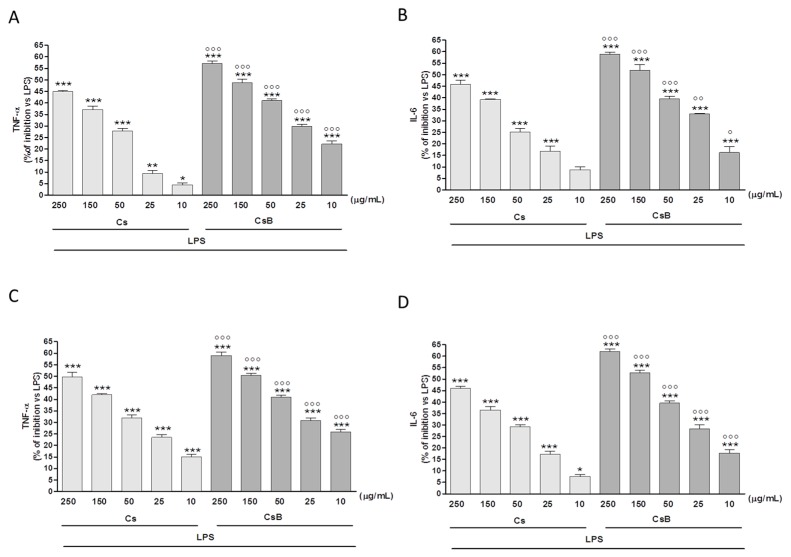
Effect of Cs and CsB (250–10 μg/mL) on LPS –induced TNF-α (Panel **A** and Panel **C**) and IL-6 (Panel **B** and Panel **D**) production in J774A.1 macrophages. TNF-α and IL-6 production was measured in the supernatants of J774A.1 cells treated with Cs and CsB (250–10 μg/mL) and LPS (1 µg/mL) for 18 h by means of ELISA. Values, mean ± s.e.m., are expressed as % of inhibition vs. J774A.1 treated with LPS alone. ***, ** and * denote *p* < 0.001, *p* < 0.01 *p* < 0.05 vs. LPS alone. °°°, °° and ° denote *p* < 0.001, *p* < 0.01 and *p* < 0.05 vs. Cs.

Macrophages exposed to Cs or CsB, significantly reduced TNF-α secretion in response to LPS administration. The effect was detectable either when Cs or CsB were added before LPS (*p* < 0.05 vs. LPS; Figure 3A) and 24 h after LPS (*p* < 0.001 vs. LPS; Figure 3C). Confirming the previously reported evidence, CsB administration showed highest inhibitory activity compared to Cs (*p* < 0.001 vs. Cs, Figure 3A,C). Similar results were obtained by evaluating the effect of the two extracts on IL-6 production. CsB was the most active either when added before (*p* < 0.001 vs. LPS and *p* < 0.05 vs. Cs; Figure 3B) and 24 h later LPS (*p* < 0.05 vs. LPS and *p* < 0.001 vs. Cs; Figure 3D).

### 2.6. Effect of Citrus Extracts on p65 NF-kB Nuclear Translocation

**Figure 4:**
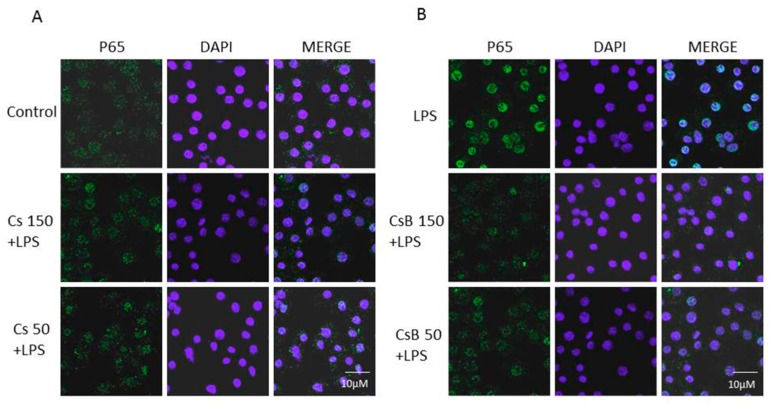
Effect of Cs and CsB (150–50 μg/mL) on p65 nuclear translocation in normal (**A**) and in inflammatory conditions (**B**) in J774A.1 macrophages. Nuclear translocation of NF-κB p65 subunit was detected using immunofluorescence assay at confocal microscopy. Scale bar, 10 µm. Blue and green fluorescence indicate localization of nucleus (DAPI) and p65 respectively. Analysis was performed by confocal laser scanning microscopy.

Macrophages respond to LPS translocating the pro-inflammatory transcription factor NF-kB into the nucleus [28]. NF-κB is a central regulator of inflammation [29]. Following p65 phosphorylation, the free NF-κB dimers translocate into the nucleus and bind to specific sequences to regulate the downstream genes expression [30]. Thus, we labelled p65 with a green fluorescence to track the influence of Cs and CsB tested at two medium concentrations (150–50 μg/mL, Figure 4A) and added 1 h before LPS (1 μg/mL, Figure 4B) on p65 NF-κB translocation. As shown in Figure 4, nuclear p65 translocation increased 15 min after LPS administration. In J774A.1 macrophages Cs or CsB exposure reduced NF-κB translocation if compared to LPS alone. Consistently with previous data CsB was able to better prevent NF-κB translocation.

### 2.7. Effect of Citrus Extracts on LPS-Induced ROS and Induced HO-1 Expression

**Figure 5:**
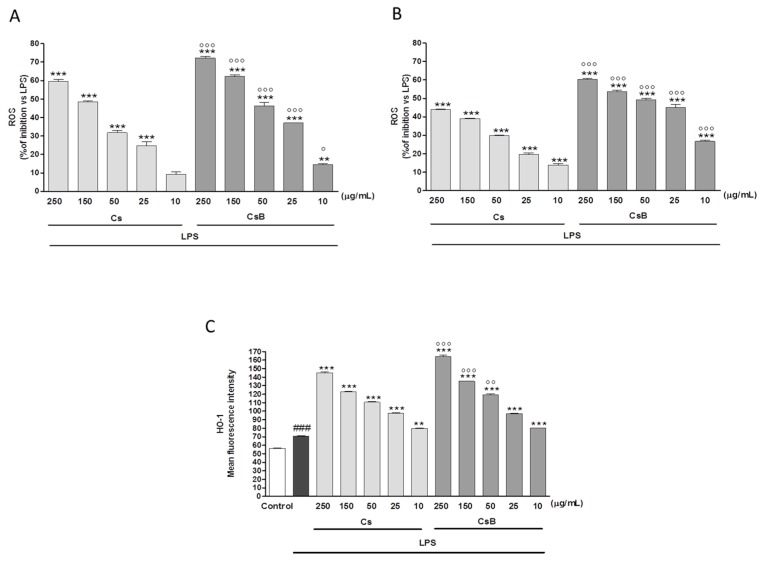
Effect of Cs and CsB (250–10 μg/mL) on LPS-induced ROS in LPS-stimulated J774A.1 macrophages (Panel **A** and Panel **B**); Effect of Cs and CsB (250–10 μg/mL) on LPS-induced HO-1 (Panel **C**) in macrophages J774A.1. Values, mean ± s.e.m., are expressed as % of inhibition vs. J774A.1 treated with LPS alone and as mean fluorescence intensity. ^###^ denotes *p* < 0.001 vs. control. *** and ** denote *p* < 0.001 and *p* < 0.01 vs. LPS alone. °°°, °° and ° denote *p* < 0.001, *p* < 0.01 and *p* < 0.05 vs. Cs.

Cs (250–10 µg/mL) significantly inhibited ROS production in a concentration range of 250–25 µg/mL both when added 1 h before (*p* < 0.001 vs. LPS; Figure 5A) and 24 h after LPS (*p* < 0.001 vs. LPS Figure 5B). In accordance with the previously reported results, CsB significantly inhibited ROS production both versus LPS and versus Cs (*p* < 0.001; Figure 5A,B) under both experimental conditions. In light of the HO-1 anti-inflammatory and cytoprotective functions [31], also under oxidative stress conditions, we evaluated whether its expression was influenced by *Citrus* extracts. While expressed at low levels in basal condition in J774A.1 macrophages, HO-1 resulted increased by LPS (*p* < 0.001 vs. control; Figure 5C). Cs further increased the enzyme expression when compared to LPS at all tested concentrations (*p* < 0.01). It’s worth noting that in this experiment, as observed before, CsB exerted a stronger effect than Cs (*p* < 0.01; Figure 5C).

### 2.8. CsB and Cs Suppress LPS-Mediated Inflammatory Response in Mouse Primary Macrophages

**Figure 6:**
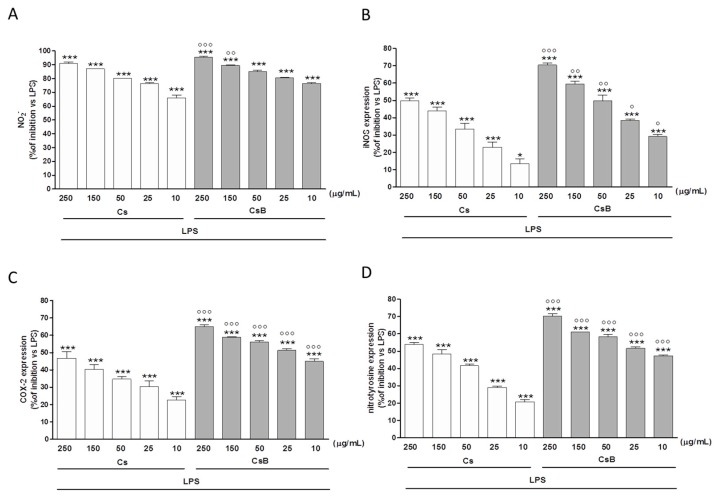
Effect of Cs and CsB (250–10 μg/mL) on NO release, evaluated as NO_2_^−^ (µM), by peritoneal macrophages stimulated with LPS (Panel **A**); Effect of Cs and CsB (250–10 μg/mL) on LPS-induced iNOS (Panel **B**); COX-2 (Panel **C**) and nitrotyrosine (Panel **D**) in peritoneal macrophages. Values, mean ± s.e.m., are expressed as % of inhibition vs. peritoneal macrophages treated with LPS alone. *** and * denotes *p* < 0.001 and *p* < 0.05 vs. LPS alone. °°°, °° and ° denote *p* < 0.001, *p* < 0.01 and *p* < 0.05 vs. Cs.

**Figure 7:**
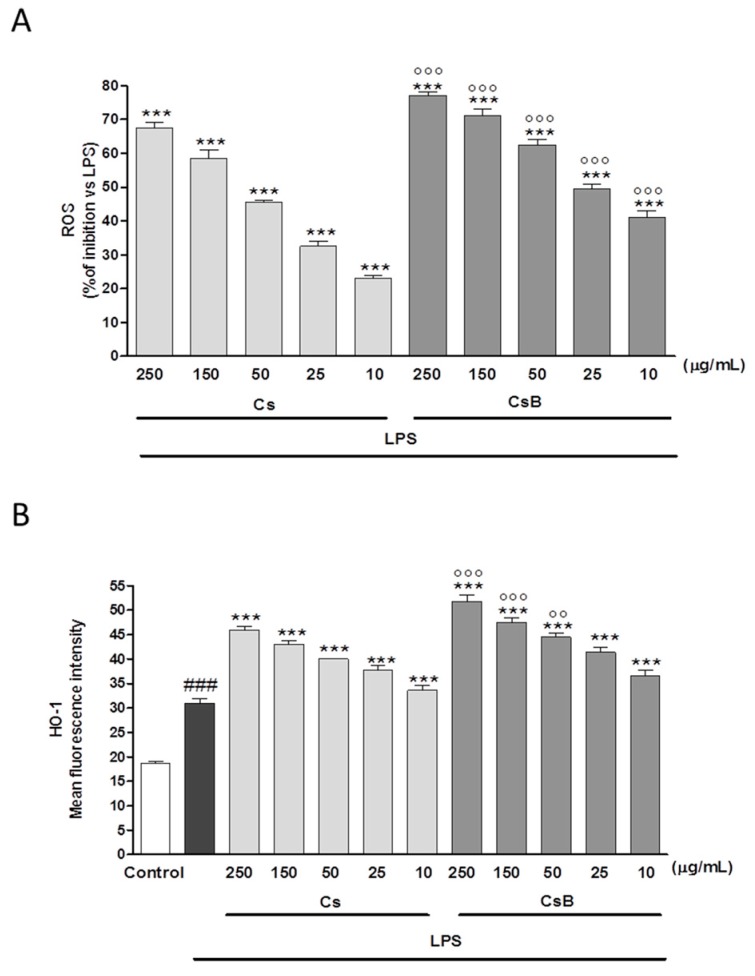
Effect of Cs and CsB (250–10 μg/mL) on LPS-induced ROS in LPS-stimulated peritoneal macrophages (Panel **A**); Effect of Cs and CsB (250–10 μg/mL) on LPS-induced HO-1 (Panel **B**) in peritoneal macrophages. Values, mean ± s.e.m., are expressed as % of inhibition vs. peritoneal macrophages treated with LPS alone. ^###^ denotes *p* < 0.001 vs. control. *** denotes *p* < 0.001 vs. LPS alone. °°° and °° denote *p* < 0.001 and *p* < 0.01 vs. Cs.

To further confirm the observed effect of CsB and Cs following LPS-induced inflammation, we evaluated NO release, iNOS and COX-2 expression, nitrotyrosine formation as well as ROS release and HO-1 expression in mouse peritoneal primary macrophages. Results indicated that CsB was more efficient than Cs in inhibiting pro-inflammatory and oxidative stress parameters also in primary murine macrophages (*p* < 0.001 vs. LPS and Cs; Figure 6A–D; Figure 7A,B).

## 3. Discussion

The antioxidant properties of flavonoids are deeply dependent on their chemical structure. Free radical quenching has been related, for example, to the ability of the flavonoids aromatic hydroxyl group to donate the hydrogen delocalizing the unpaired electron around the aromatic system [32]. In addition, chelation of the transition metal ions catalyzing the formation of free radicals has been proposed as alternative mechanism [33]. We found that the relative ratio among different compounds of *Citrus sinesis* extract changes after GI difestion and uptake. In particular, the relative increase in the amount of polymethoxyflavones (nobiletin, sinesetin and isosinensetin) could be considered responsible for the higher antioxidant and anti-inflammatory activity showed by the bioavailable fraction [34]. This observation is in accordance with the results obtained by Shin and co-workers, that revealed the pharmacodynamic improvement exerted by methoxy-substitued flavonoids in the activation of the endogenous antioxidant signalling [35]. Moreover, different studies revealed that aglycones have a greater antioxidant effect if compared with their conjugated flavonoids, probably due both to pharmacodynamic (less steric hindrance of the reactive oxydryl groups) and pharmacokinetic (increase in membranes accessibility) effects [32].

## 4. Materials and Methods

### 4.1. Reagents and Standards

Ultra-pure water (H_2_O) was obtained by a Milli-Q Direct 8 system (Millipore, Milan, Italy), methanol, acetonitrile (ACN) and formic acid (HCOOH) LC-MS grade were purchased from Sigma Aldrich (Milan, Italy). For the quantitative and qualitative analysis of flavonoids two columns were employed respectively: a Kinetex C18 150 × 4.6 mm (100 Å), packed with 2.6 µm particles, and a Kinetex C18 150 × 2.1 mm, 2.6 µm column (Phenomenex, Bologna, Italy). Both columns were protected with C18 precolumns (Phenomenex).

Flavonoids standards (diosmetin 6,8 di C-glucoside, neohesperidin, eriocitrin, isoquercetin, narirutin, diosmetin, hesperetin) and polymetoxyflavones (tangeretin) were purchased from Sigma Aldrich. For cell culture unless stated otherwise, all reagents and compounds were purchased from Sigma Chemicals Company (Milan, Italy).

### 4.2. Sample Preparation

The *Citrus sinensis* juice var. Tarocco was provided by the company “Agrumaria Corleone” (Palermo, Italy), which used fruits from plants cultivated in Sicily (Italy). In order to remove fibers, 100 mL of hand squeezed juice were centrifuged at 15,000× *g* for 15 min, then lyophilized for 24 h, by setting the condenser temperature at −52 °C and the vacuum value at 0.100 mBar. From 100 mL of *Citrus sinensis* juice, 400 mg of dry extract was obtained. The powder was extracted with MeOH and the procedure was repeated three times for the complete recovery of polyphenolic compounds. The methanolic extracts were combined and the organic solvent was removed by vacuum evaporation at 50 mBar and 40 °C. Sample was stored at 4 °C, then solubilized in methanol to a concentration of 1 mg/mL, subjected to ultrasonication and filtered prior to injection on 0.45 µm nylon membrane (Millipore).

### 4.3. Instrumentation

UHPLC analyses were performed on a Nexera UHPLC system (Shimadzu, Kyoto, Japan) consisting of a CBM-20A controller, two LC-30AD dual-plunger parallel-flow pumps, a DGU-20 A_R5_ degasser, an SPD-M20A photo diode array detector (equipped with a 2.5 µL detector flow cell volume), a CTO-20A column oven, a SIL-30AC autosampler. The UHPLC system was coupled online to an LCMS–IT-TOF mass spectrometer through an ESI source (Shimadzu). LC-MS data elaboration was performed by the LCMSsolution^®^ software (Version 3.50.346, Shimadzu).

### 4.4. UHPLC PDA Conditions

The optimal mobile phase consisted of 0.1% HCOOH/H_2_O *v/v* (A) and 0.1% HCOOH/ACN *v/v* (B). Analysis was performed in gradient elution as follows: 0–2.00 min, 10–15% B; 2–10.00 min, 15–20% B; 10–12.50 min, 20–65% B; 12.50–17.50 min, 65–75% B. Flow rate was 1.8 mL/min. Column oven temperature was set to 40 °C. Injection volume was 2 µL of extract. The following PDA parameters were applied: sampling rate, 40 Hz; detector time constant, 0.160 s; cell temperature, 40 °C. Data acquisition was set in the range 190–400 nm and chromatograms were monitored at 280 and 330 nm at the maximum absorbance of the compounds of interest.

For the quantification of flavonoids, eight compounds were selected as external standards: diosmetin 6,8-di-C-glucoside, neohesperidin, eriocitrin, isoquercetin, narirutin, diosmetin, hesperetin and tangeretin. Stock solutions (1 mg·mL^−1^) were prepared in methanol, and the calibration curves were obtained in a concentration range of 0.5–100 µg·mL^−1^ with seven concentration levels and triplicate injections of each level were run. Peak areas of each standard were plotted against corresponding concentrations (µg·mL^−1^). The amount of the compounds in the sample was expressed as milligram per gram of extract, linear regression was used to generate calibration curve, R^2^ values were ≥0.995.

### 4.5. UHPLC-ESI-IT-TOF Conditions

Mobile phases were: (A) H_2_O and (B) ACN both acidified by formic acid 0.1% *v/v*. Analysis was performed in gradient elution as follows: 0–2.50 min, 5–15% B, 2.50–10.00 min, 15–25% B, 10–12.00 min, 25–55% B, 12–14.50 min, 55–65% B, 14.50–17.00 min, 65–70% B. Flow rate was 0.5 mL/min. Column oven temperature was set to 40 °C. Injection volume was 2 µL. The UHPLC system was coupled on-line to a hybrid IT-TOF instrument, flow rate from LC was splitted 50:50 prior of the ESI source by means of a stainless steel Tee union (1/16 in, 0.15 mm bore, Valco, Houston, TX, USA). Resolution, sensitivity, and mass number calibration of the ion trap and the TOF analyzer were tuned using a standard sample solution of sodium trifluoroacetate. After the calibrant had flowed, cleaning operation of the tube and ESI probe was carried out by flowing acetonitrile (0.2 mL/min, 20 min). MS detection was operated both positive and negative ionization mode with the following parameters: detector voltage, 1.55 kV; CDL (curve desolvation line) temperature, 200 °C; block heater temperature, 200 °C; nebulizing gas flow (N_2_), 1.5 L/min; drying gas pressure, 100 kPa. Full scan MS data were acquired in the range of 200–800 *m*/*z* (ion accumulation time, 40 ms; IT, repeat = 2). MS/MS experiments were conducted in data dependent acquisition, precursor ions were acquired in the range 150–800 *m*/*z*; peak width, 3 Da; ion accumulation time, 60 ms; CID energy, 50%; collision gas, 50%; repeat = 1; execution trigger (BPC) intensity, at 95% stop level.

### 4.6. In Vitro Gastrointestinal Digestion

The assay was performed according to the procedure described by Tenore et al. [36]. GI digestion was distinguished into salivary, gastric and duodenal digestive steps. For the salivary digestion, the sample (1 g), prepared as described above, was mixed with 6 mL of artificial saliva composed of: KCl (1.2 M), KSCN (0.2 M), NaH_2_PO_4_ (0.7 M), Na_2_SO_4_ (0.4 M), NaCl (175.3 g/L, 3.0 M), NaHCO_3_ (1.0 M), urea (0.4 M) and 290 mg of α-amylase. The pH of the solution was adjusted to 6.8 with HCl 0.1 M. The mixture was introduced in a plastic bag containing 40 mL of water and homogenized in a Stomacher 80 Microbiomaster (Seward, Worthing, UK) for 3 min. Immediately, 0.5 g of pepsin (14,800 U) dissolved in HCl 0.1 M were added, the pH was adjusted to 2.0 with HCl 6 M, and then incubated at 37 °C in a Polymax 1040 orbital shaker (250 rpm, Heidolph, Schwabach, Germany) for 2 h. After the gastric digestion, the pancreatic digestion was simulated as follows: the pH was increased to 6.5 with NaHCO_3_ 0.5 M and then 5 mL of a mixture pancreatin (8.0 mg/mL) and bile salts (50.0 mg/mL; 1:1; *v/v*), dissolved in 20 mL of water, were added and incubated at 37 °C in an orbital shaker (250 rpm) for 2 h. After each step of digestion, 10 mL of the obtained extract were centrifuged at 1700× *g* and 4 °C for 1 h: before each following step, the digestion procedure was started over again. To determine the polyphenolic profile, the supernatants were extracted with an acetonitrile–water (84:16; *v/v*) mixture, and then analysed by UHPLC.

### 4.7. Cell Culture

#### 4.7.1. Caco-2 Cell Line

The human colon carcinoma cell line Caco-2 (HTB-37) was obtained from the American Type Culture Collection (LGC Promochem, Molsheim, France). Cells were cultured routinely in HEPES buffered Dulbecco’s modified Eagle’s medium (DMEM) with 4.5 g/L glucose and supplemented with 12.5% heat-decomplemented fetal calf serum (FCS), 1% nonessential amino acids, 5 mM L-glutamine, 40 U/mL penicillin, 100 µg/mL gentamycin, and 40 µg/mL streptomycin (DMEMc). Cells were maintained at 37 °C in a humidified atmosphere of CO_2_/air (5:95) and passaged every 7 days by trypsinization.

Caco-2 cells were seeded in transwells at 6 × 10^4^ cells/cm^2^ in 15 mL DMEM containing 10% FCS and cultured for was changed every 21 days. The integrity of the monolayers was evaluated by measurement of the transepithelial electrical resistance (TEER) using a Millicell-ERS device (Millipore, Zug, Switzerland) before and after the treatments. To evaluate transepithelial permeability, the medium was removed from the apical and basal sides of the cultures and replaced by 2 mL of the transport solution consisting of Hanks’ balanced salt solution (HBSS) and *Citrus sinensis* juice flavonoids deriving from gastrointestinal digestion at pH 6 or 7.4. After 4 h of incubation at 37 °C, apical and basal solutions were collected and to determine the polyphenolic profile, aliquots (5 mL) were immediately mixed with 1 mL of methanol and filtered on 0.45 µm Millex-HV filter units. Samples were stored at −20 °C until HPLC analysis.

#### 4.7.2. J774A.1 Macrophage Cell Line

J774A.1 murine monocyte macrophage cell line (American Type Culture Collection, Rockville, MD, USA), was grown adherent to Petri dishes with Dulbecco’s modified Eagle’s medium (DMEM) supplemented with 10% foetal calf serum (FCS), 25 mM HEPES, 2 mM glutamine, 100 μ/mL penicillin and 100 mg/mL streptomycin at 37 °C in a 5% CO_2_ atmosphere.

#### 4.7.3. Primary Murine Peritoneal Macrophages

Female C57BL/6 mice (6–8 weeks; Harlan Laboratories, Udine, Italy) were fed a standard chow diet and housed under specific pathogen-free conditions at the University of Salerno Department of Pharmacy. All animal experiments were performed under protocols that followed the Italian and European Community Council for Animal Care (DL No. 116/92). Peritoneal cells were harvested by means of peritoneum lavage with 5 mL of EDTA 0.5 mM plated and allowed to adhere for 2 h at 37 °C in a 5% CO_2_ atmosphere. Subsequently, non-adherent cells were removed and RPMI 1640 medium with 10% FBS was added, as previously reported [37].

### 4.8. Experimental Procedures

Macrophages, both J774A.1 and primary macrophages, were incubated with raw *Citrus sinensis* (Cs) or bioavailable *Citrus sinensis* (CsB) (250–10 μg/mL) alone for 1 h and then simultaneously to lipopolysaccharide from *E. coli* (LPS; 10 µg/mL) for the time described below. In some experiments, in order to evaluate the effect Cs and CsB after inflammation induction, J774A.1 macrophages were firstly treated with LPS and then with Cs or CsB for further 24 h, always in presence of LPS, before the evaluation of pro-inflammatory and pro-oxidant parameters.

### 4.9. Antiproliferative Activity

Macrophages were plated on 96-well plates (8 × 10^3^/well and 5 × 10^4^/well respectively) and treated with serial dilutions of Cs or CsB (250–10 μg/mL) for 24, 48 and 72 h. Cell viability was assessed using the MTT assay as previously reported [38]. Briefly, 25 mL of MTT (5 mg/mL) were added and cells were incubated for an additional 3 h. Thereafter, cells were lysed and the dark blue crystals dissolved with 100 mL of a solution containing 50% (*v*/*v*) *N*,*N*-dimethylformamide, 20% (*w*/*v*) SDS with an adjusted pH of 4.5. The optical density (OD) of each well was measured with a microplate spectrophotometer (Titertek Multiskan MCC/340-DASIT, Firenze, Italy) equipped with a 620 nm filter. Cell viability in response to treatment with Cs and CsB, was calculated as: % dead cells = ((OD treated/OD control) × 100).

### 4.10. Measurement of Intracellular Reactive Oxygen Species (ROS)

ROS formation was evaluated by means of the probe 2′,7′-dichlorofluorescin-diacetate (H_2_DCF-DA) as previously reported [39]. H_2_DCF-DA is a non-fluorescent permeant molecule that passively diffuses into cells, where the acetates are cleaved by intracellular esterases to form H_2_DCF and thereby trapped it within the cell. In the presence of intracellular ROS, H_2_DCF is rapidly oxidized to the highly fluorescent 2′,7′-dichlorofluorescein (DCF). Briefly, J774A.1 (3.0 × 10^5^/well) and mouse peritoneal macrophages (3.0 × 10^5^/well) were plated into 24-well plates and treated with Cs, or CsB (250–10 μg/mL) and LPS as described above, for 24 h. Cells were then collected, washed twice with phosphate buffer saline (PBS) buffer and then incubated in PBS containing H_2_DCF-DA (10 µM) at 37 °C. After 45 min, cells fluorescence was evaluated using a fluorescence-activated cell sorting (FACSscan; Becton Dickinson, Durham, NC, USA) and elaborated with Cell Quest software (version 6.0, Becton Dickinson, Durham, NC, USA).

### 4.11. Immunofluorescence Analysis with Confocal Microscopy in Macrophages J774A.1

For immunofluorescence assay, J774A.1 cells (3 × 10^5^/well) were seeded on coverslips in 12 well plate and treated with Cs or CsB (150–50 µg/mL) and LPS (1 µg/mL), as described above, for 20 min in order to detect NF-kB nuclear translocation.

Cells were then fixed with 4% paraformaldehyde in PBS for 15 min and permeabilized with 0.1% saponin in PBS for 15 min. After blocking with BSA and PBS for 1 h, macrophages were incubated with Rabbit anti-phospho p65 antibody (Santa Cruz Biotechnologies, Dallas, TX, USA) for 1 h at room temperature. The slides were then washed with PBS for three times and fluorescein-conjugated secondary antibody (FITC) was added for 1 h, DAPI was used for counterstaining of nuclei. Coverslips were finally mounted in mounting medium and fluorescent images were taken under the Laser Confocal Microscope (Leica TCS SP5, Wetzlar, Germany) [40].

### 4.12. Nitrite Determination and Cytofluorimetry Evaluation of iNOS, COX-2 and HO-1 Expression and Nitrotyrosine Formation in J774A.1

Macrophages J774A.1 were plated in 96 well plate (5.0 × 10^4^/dish) and allowed to adhere for 4 h. Thereafter, the medium was replaced with fresh medium alone or containing serial dilutions of Cs or CsB (250–10 μg/mL) alone and then co-exposed to LPS (1 μg/mL) for further 24 h to detect nitrite (NO_2_^−^), iNOS, COX-2 and HO-1 expression and nitrotyrosine formation. NO production was measured as NO_2_^−^, index of NO released by cells, in the culture medium 24 h after LPS stimulation by Griess reaction, as previously reported [41].

Briefly, 100 mL of cell culture medium were mixed with 100 mL of Griess reagent~equal volumes of 1% (*w/v*) sulphanilamide in 5% (*v/v*) phosphoric acid and 0.1% (*w/v*) naphtylethylenediamine-hydrogen chloride (HCl) and incubated at room temperature for 10 min, and then the absorbance was measured at 550 nm in a microplate reader Titertek (Dasit, Cornaredo, Milan, Italy). The amount of NO_2_^−^, as μM concentration, in the samples was calculated by a sodium NO_2_^−^ standard curve. In order to evaluated the iNOS, COX-2 and HO-1 expression and nitrotyrosine formation were evaluate by cytofluorimetry, cells were then collected, washed twice PBS and then incubated in fixing solution for 20 min at 4 °C and then incubated in Fix Perm Solution for 30 min at 4 °C. Anti-iNOS, anti-COX-2, anti-HO-1 and anti-nitrotyrosine (Millipore) were then added for further 30 min. The secondary antibody was added in Fix solution and cells fluorescence was evaluated using a fluorescence-activated cell sorting (FACSscan; Becton Dickinson) and elaborated with Cell Quest software as previously reported [40,41].

### 4.13. TNF-α and IL-6 Determination in Macrophages J774A.1

TNF-α and IL-6 concentrations in J774A.1 treated with Cs or CsB (250–10 μg/mL) and LPS (1 μg/mL) for 18 h, as previously described, were assessed by an Enzyme-Linked Immuno Sorbent Assay (ELISA) assay by using a commercial kit, for murine TNF-a or IL-6, according to manufacturer’s instruction (e-Biosciences, San Diego, CA, USA).

### 4.14. NO Determination and iNOS, COX-2, Nitrotyrosine, ROS and HO-1 Detection in Mouse Peritoneal Macrophages

Mouse peritoneal macrophages were plated into 96-well plates (3.5 × 10^4^/well). Peritoneal macrophages were allowed to adhere for 24 h at 37 °C in a 5% CO_2_ atmosphere before experiments. Thereafter the medium was replaced with fresh medium and cells were treated with Cs and CsB (250–10 μg/mL) then exposed simultaneously to LPS (1 µg/mL) as reported above. NO, iNOS, COX-2, nitrotyrosine, ROS and HO-1 and formation evaluation was detected as reported for macrophages.

### 4.15. Data Analysis

Data are reported as mean ± standard error mean (s.e.m.) values of at least three independent experiments. Statistical analysis was performed by analysis of variance test, and multiple comparisons were made by Bonferroni’s test. A *p*-value < 0.05 was considered as significant.

## 5. Conclusions

In the present study, we investigated the antioxidant and anti-inflammatory effect of bioavailable flavonoids from *Citrus sinensis* extract. Bioavailability studies were carried out by simulated GI digestion and Caco-2 cells permeation model. Data highlighted how most of the flavonoids are already bioavailable after the salivary and gastric stages, whereas the intestinal environment is mainly responsible for their degradation, in accordance with previous studies [23]. Moreover, flavonoids patterns deriving from intestinal digestion are deeply modified by cellular uptake, as simulated by the use of Caco-2 cell permeation assay. Previous studies emphasize that the reason for the overestimation of the antioxidant potential of flavonoids-rich foods comes from the differences between chemical composition of the intestinal digesta and its bioavailability [26]. Thus, in this work, the bioavailable fraction was compared to the original extract, showing higher antioxidant and anti-inflammatory potential at the same concentration. It must be considered that, although the bioavailable fraction represents only the 8.3% of the total flavonoids fraction, it maintains a remarkable antioxidant activity also at the lowest concentration used (10 µg/mL), with a comparable effect to the 5- or 15-fold more concentrated raw extract. At the best of our knowledge, this is the first study investigating the chemical composition of the bioavailable fraction of Citrus flavonoids, exploring its antioxidant and anti-inflammatory potential. The results obtained further support the belief in health benefits deriving from *Citrus sinensis* juice, that were mainly based on the antioxidant protection provided in vitro, revealing the positive rather than negative influence of digestion processes. Further in vivo studies are necessary to explore the bioavailability of the Citrus flavonoids along with their antioxidant and anti-inflammatory effects, but in vitro models have been often well-correlated to animal and human studies [42].

## Supplementary Materials

Supplementary materials are available online.
