# Supplementary material for: Bioavailable Citrus sinensis Extract: Polyphenolic Composition and Biological Activity
Source: Molecules. 2017 Apr 15;22(4):623. doi: 10.3390/molecules22040623 (PMC6154582; doi:10.3390/molecules22040623)
Supplement: Supplementary file 1 [file molecules-22-00623-s001.pdf]

Table S1

Peak	*k'*	$[M-H]^-$	$[M-H]^+$	MS^2 *m/z*	Error (ppm)	Regression Curve	R^2	LOD (ppm)	LOQ (ppm)	Extract flavonoids *	***In vitro* gastrointestinal digestion** Salivary *	Gastric *	Intestinal *	Dialysis *	Compound
1	2.64	593.1518	–	353.0691	1.18	y = 1.2604E^{-06} x – 1.0047E^{-03}	0.9981	0.8921	1.5622	49.11 ± 2.87	36.86 ± 0.42	34.38 ± 0.20	8.01 ± 0.13	2.53 ± 0.01	vicenin-2
2	3.03	623.1628	–	383.0791	1.60	y = 1.2604E^{-06} x – 1.0047E^{-03}	0.9981	0.8921	1.5622	32.77 ± 0.47	26.24 ± 0.80	24.58 ± 0.29	4.65 ± 0.12	1.71 ± 0.02	lucenin-2 4'-methyl ether
3	4.56	609.1478	–	301.0382	2.79	y = 1.0012E^{-06} x – 2.2930E^{-03}	0.9953	0.6987	1.2235	9.75 ± 2.97	8.31 ± 0.88	7.81 ± 0.04	1.98 ± 0.10	0.95 ± 0.08	neohesperidin
4	6.24	595.1692	–	288.9530	4.03	y = 1.2657E^{-06} x – 2.3642 E^{-04}	0.9962	0.8976	1.5718	5.86 ± 0.11	5.27 ± 0.07	4.98 ± 0.12	1.31 ± 0.12	0.57 ± 0.02	eriocitrin
5	6.72	463.0890	–	301.0353	1.73	y = 8.9337E^{-07} x – 1.7211E^{-03}	0.9983	0.6322	1.1071	4.06 ± 0.94	4.02 ± 0.45	3.57 ± 0.08	0.76 ± 0.09	0.36 ± 0.01	isoquercitrin
6	7.44	579.1835	–	271.0636	1.35	y = 1.0043E^{-06} x – 3.2128E^{-04}	0.9996	0.7098	1.2429	55.14 ± 1.97	43.01 ± 0.93	40.25 ± 0.55	13.34 ± 1.62	2.93 ± 0.09	narirutin
7	8.49	607.1317	–	300.0320	1.98	y = 4.7437E^{-07} x + 1.5354E^{-04}	0.9996	0.7458	1.3060	2.72 ± 0.62	2.26 ± 0.24	2.15 ± 0.05	0.55 ± 0.02	0.26 ± 0.03	neodiosmin
8	9.38	609.1738	–	301.0736	3.28	y = 6.4061E^{-07} x – 1.9685E^{-03}	0.9966	0.4657	0.8155	207.65 ± 1.77	184.81 ± 2.76	176.51 ± 1.05	30.94 ± 3.49	10.73 ± 0.07	hesperidin
9	13.73	593.1895	–	285.0763	3.20	y = 6.4061E^{-07} x – 1.9685E^{-03}	0.9966	0.4657	0.8155	3.55 ± 0.15	3.37 ± 0.07	3.12 ± 0.20	0.71 ± 0.13	0.31 ± 0.03	didymin
10	16.55	–	373.1236	343.0805	3.48	y = 4.2086E^{-07} x + 9.9601E^{-04}	0.9975	0.0540	0.1018	3.62 ± 1.25	2.79 ± 0.21	2.64 ± 0.03	1.81 ± 0.13	1.64 ± 0.02	isosinensetin
11	16.73	–	403.1371	373.0918	-3.97	y = 4.2086E^{-07} x + 9.9601E^{-04}	0.9975	0.0540	0.1018	2.35 ± 0.97	0.96 ± 0.09	–	–	–	hexamethoxyflavone
12	16.88	–	373.1268	312.0990	3.49	y = 4.2086E^{-07} x + 9.9601E^{-04}	0.9975	0.1343	0.2644	7.64 ± 0.99	6.42 ± 0.60	6.03 ± 0.28	3.96 ± 0.23	3.57 ± 0.04	sinensetin
13	17.00	–	403.1362	373.0923	-0.25	y = 4.2086E^{-07} x + 9.9601E^{-04}	0.9975	0.1343	0.2644	2.66 ± 0.78	–	–	–	–	hexamethoxyflavone *isomer*
14	17.09	–	403.1331	373.0936	0.50	y = 4.2086E^{-07} x + 9.9601E^{-04}	0.9975	0.2981	0.5220	21.22 ± 1.15	21.04 ± 0.31	20.12 ± 0.71	11.51 ± 0.72	9.42 ± 0.05	nobiletin
15	17.23	–	343.1166	282.0876	-2.91	y = 4.2086E^{-07} x + 9.9601E^{-04}	0.9975	0.2981	0.5220	2.82 ± 1.37	2.40 ± 0.10	1.98 ± 0.03	–	–	tetramethyl-*o*-isoscutellarein
16	17.35	–	433.1472	403.1019	-4.85	y = 4.2086E^{-07} x + 9.9601E^{-04}	0.9975	0.0445	0.0799	4.06 ± 1.49	3.75 ± 0.54	2.24 ± 0.06	0.85± 0.04	–	heptamethoxyflavone
17	17.46	–	373.1238	343.0809	4.02	y = 4.2086E^{-07} x + 9.9601E^{-04}	0.9975	0.0445	0.0799	5.54 ± 0.79	4.26 ± 0.66	2.93 ± 0.01	0.74 ± 0.04	–	tangeretin
18	17.97	–	389.1215	359.0741	2.06	y = 4.2086E^{-07} x + 9.9601E^{-04}	0.9975	0.0618	0.1091	–	–	–	–	–	hydroxypentamethoxyflavone
19	18.32	–	419.1318	389.0866	-4.53	y = 4.2086E^{-07} x + 9.9601E^{-04}	0.9975	0.0618	0.1091	–	–	–	–	–	3-hydroxynobiletin

*: *The amount of the compounds was expressed as milligram per gram of extract. Data are reported as mean ± relative standard deviation (RSD %) values of at least three independent experiments.*

LOD: *limit of detection*

LOQ: *limit of quantitation*

R^2: *linear correlation coefficient*

k': *capacity factor* $\left(\frac{Tr - T0}{T0}\right)$; *Tr: retention time; T0: dead time.*

Figure S1

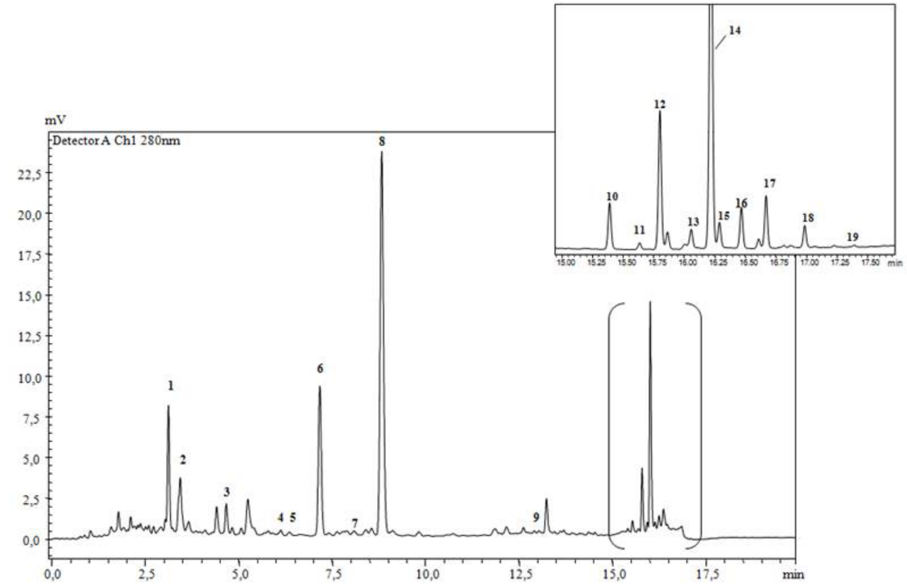

mV
Detector A Ch1 280nm
22,5
20,0
17,5
15,0
12,5
10,0
7,5
5,0
2,5
0,0
0,0
2,5
5,0
7,5
10,0
12,5
15,0
17,5
min
1
2
3
4
5
6
7
8
9
10
11
12
13
14
15
16
17
18
19
